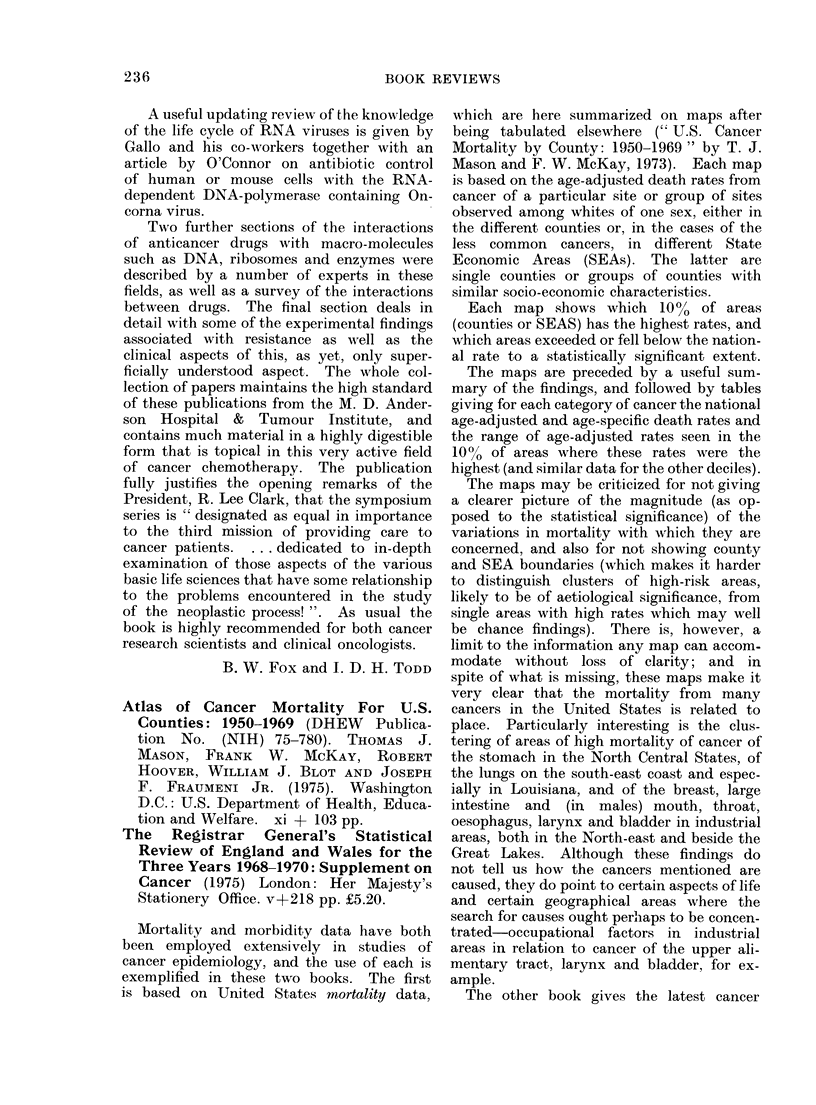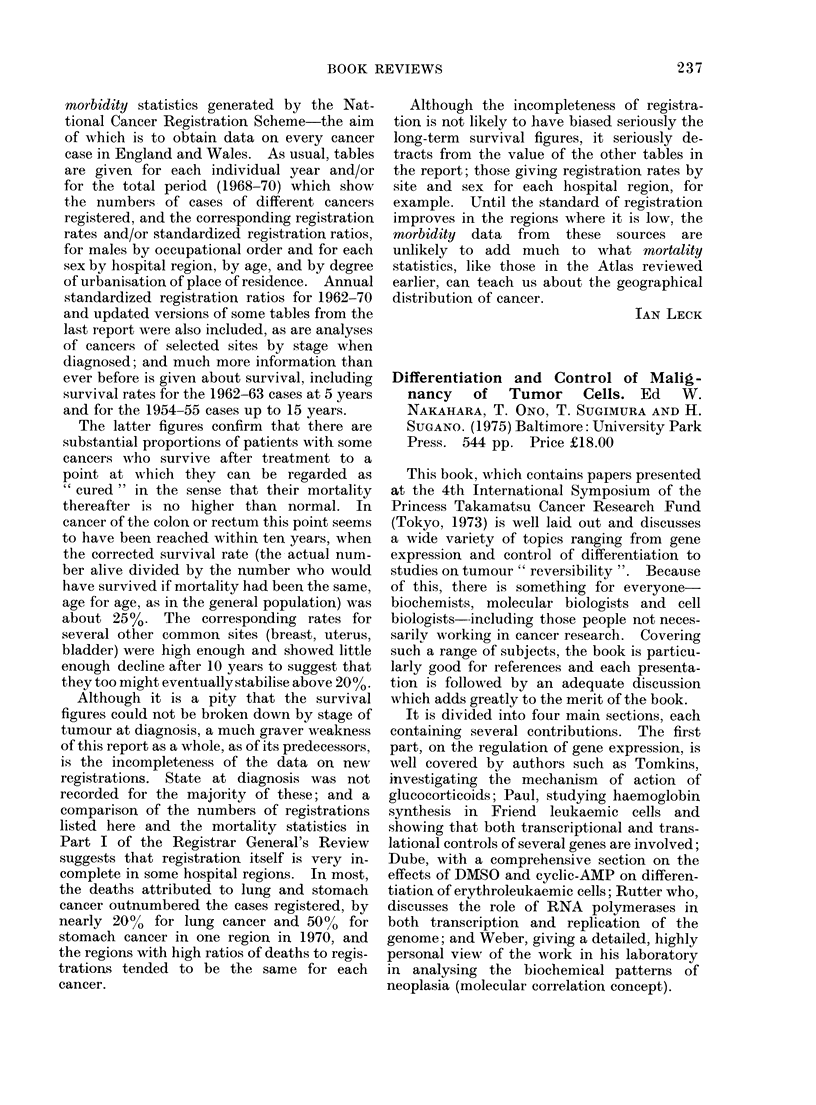# Atlas of Cancer Mortality For U.S. Counties: 1950-1969

**Published:** 1976-02

**Authors:** Ian Leck


**Atlas of Cancer Mortality For U.S. Counties: 1950–1969** (DHEW Publication No. (NIH) 75–780). Thomas J. Mason, Frank W. McKay, Robert Hoover, William J. Blot and Joseph F. Fraumeni Jr. (1975). Washington D.C.: U.S. Department of Health, Education and Welfare. xi + 103 pp.

**The Registrar General's Statistical Review of England and Wales for the Three Years 1968–1970: Supplement on Cancer** (1975) London: Her Majesty's Stationery Office. v+218 pp. £5.20.

Mortality and morbidity data have both been employed extensively in studies of cancer epidemiology, and the use of each is exemplified in these two books. The first is based on United States *mortality* data, which are here summarized on maps after being tabulated elsewhere (" U.S. Cancer Mortality by County: 1950–1969 " by T. J. Mason and F. W. McKay, 1973). Each map is based on the age-adjusted death rates from cancer of a particular site or group of sites observed among whites of one sex, either in the different counties or, in the cases of the less common cancers, in different State Economic Areas (SEAs). The latter are single counties or groups of counties with similar socio-economic characteristics.

Each map shows which 10% of areas (counties or SEAS) has the highest rates, and which areas exceeded or fell below the national rate to a statistically significant extent.

The maps are preceded by a useful summary of the findings, and followed by tables giving for each category of cancer the national age-adjusted and age-specific death rates and the range of age-adjusted rates seen in the 10% of areas where these rates were the highest (and similar data for the other deciles).

The maps may be criticized for not giving a clearer picture of the magnitude (as opposed to the statistical significance) of the variations in mortality with which they are concerned, and also for not showing county and SEA boundaries (which makes it harder to distinguish clusters of high-risk areas, likely to be of aetiological significance, from single areas with high rates which may well be chance findings). There is, however, a limit to the information any map can accommodate without loss of clarity; and in spite of what is missing, these maps make it very clear that the mortality from many cancers in the United States is related to place. Particularly interesting is the clustering of areas of high mortality of cancer of the stomach in the North Central States, of the lungs on the south-east coast and especially in Louisiana, and of the breast, large intestine and (in males) mouth, throat, oesophagus, larynx and bladder in industrial areas, both in the North-east and beside the Great Lakes. Although these findings do not tell us how the cancers mentioned are caused, they do point to certain aspects of life and certain geographical areas where the search for causes ought perhaps to be concentrated—occupational factors in industrial areas in relation to cancer of the upper alimentary tract, larynx and bladder, for example.

The other book gives the latest cancer

*morbidity* statistics generated by the Nattional Cancer Registration Scheme—the aim of which is to obtain data on every cancer case in England and Wales. As usual, tables are given for each individual year and/or for the total period (1968–70) which show the numbers of cases of different cancers registered, and the corresponding registration rates and/or standardized registration ratios, for males by occupational order and for each sex by hospital region, by age, and by degree of urbanisation of place of residence. Annual standardized registration ratios for 1962–70 and updated versions of some tables from the last report were also included, as are analyses of cancers of selected sites by stage when diagnosed; and much more information than ever before is given about survival, including survival rates for the 1962–63 cases at 5 years and for the 1954–55 cases up to 15 years.

The latter figures confirm that there are substantial proportions of patients with some cancers who survive after treatment to a point at which they can be regarded as " cured " in the sense that their mortality thereafter is no higher than normal. In cancer of the colon or rectum this point seems to have been reached within ten years, when the corrected survival rate (the actual number alive divided by the number who would have survived if mortality had been the same, age for age, as in the general population) was about 25%. The corresponding rates for several other common sites (breast, uterus, bladder) were high enough and showed little enough decline after 10 years to suggest that they too might eventually stabilise above 20%.

Although it is a pity that the survival figures could not be broken down by stage of tumour at diagnosis, a much graver weakness of this report as a whole, as of its predecessors, is the incompleteness of the data on new registrations. State at diagnosis was not recorded for the majority of these; and a comparison of the numbers of registrations listed here and the mortality statistics in Part I of the Registrar General's Review suggests that registration itself is very incomplete in some hospital regions. In most, the deaths attributed to lung and stomach cancer outnumbered the cases registered, by nearly 20% for lung cancer and 50% for stomach cancer in one region in 1970, and the regions with high ratios of deaths to registrations tended to be the same for each cancer.

Although the incompleteness of registration is not likely to have biased seriously the long-term survival figures, it seriously detracts from the value of the other tables in the report; those giving registration rates by site and sex for each hospital region, for example. Until the standard of registration improves in the regions where it is low, the *morbidity* data from these sources are unlikely to add much to what *mortality* statistics, like those in the Atlas reviewed earlier, can teach us about the geographical distribution of cancer.

IAN LECK